# Near real-time monitoring of HIV transmission hotspots from routine HIV genotyping: an implementation case study

**DOI:** 10.1016/S2352-3018(16)00046-1

**Published:** 2016-04-07

**Authors:** Art F. Y. Poon, Réka Gustafson, Patricia Daly, Laura Zerr, S. Ellen Demlow, Jason Wong, Conan K Woods, Robert S. Hogg, Mel Krajden, David Moore, Perry Kendall, Julio S. G. Montaner, P. Richard Harrigan

**Affiliations:** 1 BC Centre for Excellence in HIV/AIDS, Vancouver, BC, Canada; 2 Department of Medicine, University of British Columbia, Vancouver, BC, Canada; 3 Vancouver Coastal Health Authority, Vancouver, BC, Canada; 4 BC Centre for Disease Control, Vancouver, BC, Canada; 5 Faculty of Health Sciences, Simon Fraser University, Burnaby, BC, Canada; 6 Department of Pathology and Laboratory Medicine, University of British Columbia, Vancouver, BC, Canada; 7 Office of the Provincial Health Officer, Ministry of Health, Government of British Columbia, Victoria, BC, Canada

## Abstract

**Background:**

Due to the rapid evolution of HIV, infections with similar genetic sequences are likely to be related by recent transmission events. Clusters of related infections can represent subpopulations with high rates of HIV transmission. Here we describe the implementation of an automated “near real-time” system using clustering analysis of routinely collected HIV resistance genotypes to monitor and characterize HIV transmission hotspots in British Columbia (BC).

**Methods:**

A monitoring system was implemented on the BC Drug Treatment Database, which currently holds over 32000 anonymized HIV genotypes for nearly 9000 residents of BC living with HIV. On average, five to six new HIV genotypes are deposited in the database every day, which triggers an automated re-analysis of the entire database. Clusters of five or more individuals were extracted on the basis of short phylogenetic distances between their respective HIV sequences. Monthly reports on the growth and characteristics of clusters were generated by the system and distributed to public health officers.

**Findings:**

In June 2014, the monitoring system detected the expansion of a cluster by 11 new cases over three months, including eight cases with transmitted drug resistance. This cluster generally comprised young men who have sex with men. The subsequent report precipitated an enhanced public health follow-up to ensure linkage to care and treatment initiation in the affected subpopulation. Of the nine cases associated with this follow-up, all had already been linked to care and five cases had started treatment. Subsequent to the follow-up, three additional cases started treatment and the majority of cases achieved suppressed viral loads. Over the following 12 months, 12 new cases were detected in this cluster with a marked reduction in the onward transmission of drug resistance.

**Interpretation:**

Our findings demonstrate the first application of an automated phylogenetic system monitoring a clinical database to detect a recent HIV outbreak and support the ensuing public health response. By making secondary use of routinely collected HIV genotypes, this approach is cost-effective, attains near realtime monitoring of new cases, and can be implemented in all settings where HIV genotyping is the standard of care.

**Funding:**

This work was supported by the BC Centre for Excellence in HIV/AIDS and by grants from the Canadian Institutes for Health Research (CIHR HOP-111406, HOP-107544), the Genome BC, Genome Canada and CIHR Partnership in Genomics and Personalized Health (Large-Scale Applied Research Project HIV142 contract to PRH, JSGM, and AFYP), and by the US National Institute on Drug Abuse (1-R01-DA036307-01, 5-R01-031055-02, R01-DA021525-06, and R01-DA011591).

## Introduction

Phylogenetic clustering uses virus sequence diversity to investigate “hotspots” of rapid transmission^1^. For HIV, this approach has been facilitated by the adoption of routine genotyping for antiretroviral resistance as standard of care for clinical management of HIV infection^2,3^. A phylogeny is a tree-based representation of how populations are related through common ancestors. For rapidly-evolving viruses such as HIV^4^, the phylogeny can resemble the transmission history of the virus^5^, although they will not necessarily be congruent because of the extensive genetic diversity of the virus within each host^6^. Thus, a cluster of HIV sequences from different infections that retain a high degree of genetic similarity are likely to be related by recent transmission events^7^. Phylogenetic clusters generated from routine HIV genotype data can be informative even though these sequences tend to target a relatively conserved *pol* region of the HIV genome that encodes the primary targets of antiretroviral drug regimens, protease and reverse transcriptase (RT)^8^. Thus, a number of studies have used phylogenetic clustering for a one-time analysis of HIV *pol* datasets to retrospectively characterize potential correlates of high transmission rates of HIV, such as treatment history, stage of infection, and routes of transmission^9-12^.

Because HIV sequence-based genotypes are constantly accumulating at centers of HIV treatment, there is growing interest in utilizing this resource to inform real-time HIV prevention and control measures^13-15^. The BC Centre for Excellence in HIV/AIDS (CFE) is responsible for all HIV genotyping and distribution of antiretroviral therapy (ART) for all BC residents living with HIV, provided at no cost to the patient. Since 2013, the CFE has implemented and utilized an automated system for monitoring HIV transmission hotspots in the province by the phylogenetic clustering analysis of routine HIV genotype data. Combined with a short turn-around for sample processing, this system can provide “near real-time” information on the growth of phylogenetic clusters. We describe our implementation of a phylogenetic monitoring system in BC, and present a case study of an outbreak of transmitted HIV drug resistance detected by the system, which precipitated a formal outbreak investigation and enhanced public health follow-up of the subpopulation affected by the outbreak.

## Methods

### Population database

All data from the BC Drug Treatment Program (DTP) at the CFE is stored on site in a secure Oracle database (enterprise version 11g). The monitoring system was developed around a cached database query that integrates de-identified clinical, demographic, epidemiological and genetic data for all individuals enrolled in the DTP. The clinical data included: the sample collection dates; date of initiating ART; plasma viral loads (HIV RNA copies/mL); and HIV drug resistance levels as predicted by the VircoTYPE algorithm^16^. Dates of HIV seroconversion were imputed using the method described in Poon *et al*.^11^ (Appendix, p.1). Demographic data included gender, birth year, forward sortation area (first three digits of the Canadian postal code) of the physician office that requisitioned the laboratory test, and date of mortality when applicable^17^. Epidemiological data comprised the following risk factors: having ever used injection drugs; males self-identifying as homosexual or bisexual (men who have sex with men, MSM); and having received blood (transfusion) products. As of October 8, 2015, there were *n*=32505 HIV genotype records produced by the CFE Laboratory Program for 8839 people living with HIV – over half the estimated prevalence of HIV in BC (12981 persons in 2014). The majority (*n*=27850, 86%) of genotypes covered 1497 bp of HIV *pol* encoding protease and the first 400 codons of reverse transcriptase (RT); the remaining genotypes were derived from one or more shorter amplicons (Appendix, p.1). Transmitted drug resistance was inferred at the individual level when an HIV genotype sampled prior to initiating ART carried resistance to one or more drug classes; this does not account for people who may have started therapy outside of BC or had participated in clinical trials.

### Data analysis

The monitoring system queries the DTP database every hour. Whenever new genotype records are detected, the entire contents of the cached query are downloaded to a secure workstation. Patient identifiers are automatically replaced with random hexadecimal strings with every download to break linkage with the database. HIV genotypes are re-aligned as amino acid sequences against the HXB2 *pol* reference sequence (K03455) using a pairwise alignment algorithm^18^. Codons associated with the World Health Organization HIV drug resistance mutations surveillance list^19^ and insertions relative to HXB2 are excluded from the alignment. 100 replicate “bootstrap” alignments are generated by randomly sampling codons with replacement, and transferred to an in-house computing cluster for phylogenetic reconstruction in parallel by approximate maximum likelihood^20^ (Appendix, p.2).

Phylogenetic clusters, defined at a minimum of five members, are assembled from pairs of individuals that fulfill the following clustering criteria in a minimum of 50 out of 100 replicate phylogenies: (1) the total path length separating tips in the phylogeny (patristic distance) is below a cutoff of 0.02 expected nucleotide substitutions per site^11^; (2) the tips correspond to HIV sequences from different individuals, and; (3) at least one of the sequences is derived from the individual's earliest available sample. We use a custom tree traversal algorithm implemented in Python to efficiently extract patristic distances^11^ (Appendix, p.3). To recognize clusters between analyses in the absence of fixed patient identifiers, we use the baseline sample collection dates of its first five members to index the cluster against a persistent register (Appendix, p.3-4). Clusters are annotated with clinical and demographic variables, and visualized using a force-directed layout algorithm in GraphViz with forces proportional to the phylogenetic distance between members. Reports are generated by populating a LaTeX template document with cluster statistics and diagrams with the Jinja2 templating engine (http://jinja2.pocoo.org) in Python; a mock-up report is provided in the Appendix (p.7-17).

### Role of the funding source

The funders of the study had no role in study design, data collection, data analysis, data interpretation, or writing of the report. The corresponding author had full access to all the data in the study and had final responsibility for the decision to submit for publication.

## Results

**Figure 1:**
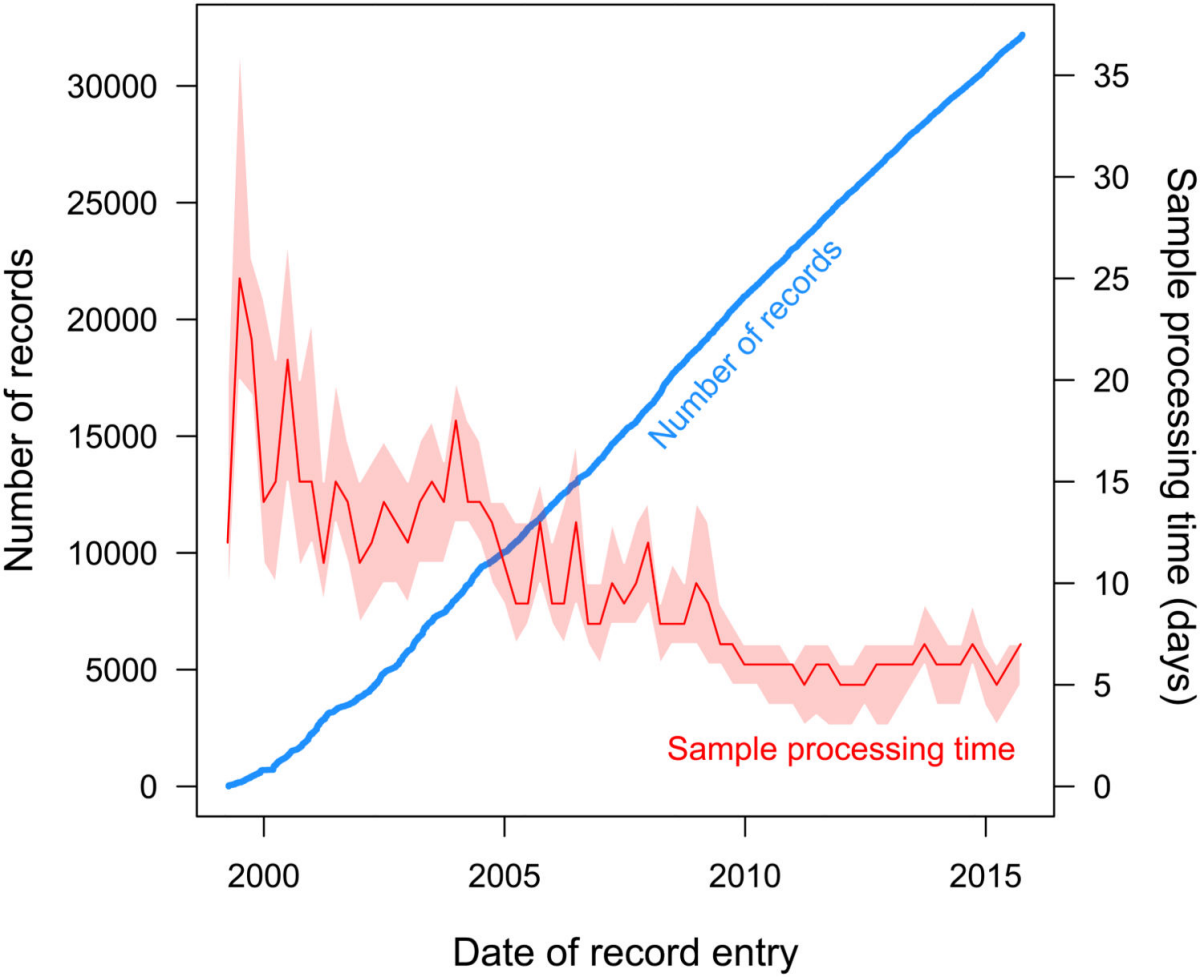
Routine HIV resistance genotyping The blue trend line represents the accumulation of HIV genotypes in the BC Drug Treatment Program (DTP) population database since the inception of the Laboratory Program at the BC Centre for Excellence in HIV/AIDS (CFE). To date, over 32,000 HIV genotypes have been deposited in the DTP database. The red trend line indicates the quarterly median (interquartile range indicated by shaded region) of sample processing times from test requisition to the date of entry of HIV genotype records into the population database.

Since April 1999, the BC DTP population database has consistently accumulated an average of 5.7 HIV genotypes every day from the CFE Laboratory Program (Figure 1). In total, over 32000 genotypes have been produced for over 8800 persons living with HIV in BC. The sample processing time, defined as the number of days between the date of a HIV genotype test requisition and the date that the test result was uploaded into the DTP database, has declined significantly since the inception of program from a median of 14 (interquartile range, IQR 11-19) days prior to 2005, to a median of 6 (IQR 4-7) days from 2010 onwards (Figure 1). Furthermore, current BC treatment guidelines stipulate that an HIV genotype test is automatically performed on the residual blood plasma from the baseline viral load test for every new HIV diagnosis. Based on data from the Seek and Treat for Optimal Prevention of HIV/AIDS (STOP) cohort in BC^22^, the median delay from HIV diagnosis to viral load testing was 26 (IQR 13-104) days.

Monthly and quarterly reports on the growth and characteristics of active clusters being tracked by our monitoring system have been distributed by the CFE since February 2014 to the BC Centre for Disease Control (BCCDC) and medical health officers at the five regional health authorities of British Columbia. A phylogenetic cluster is considered active if one or more new cases associated with the cluster have appeared in the DTP database within the reporting period. These reports were reviewed and discussed at monthly meetings between knowledge users in public health and laboratory staff from the CFE. As of October 8, 2015, a total of 218 phylogenetic clusters, each comprising five or more individuals, have been detected and tracked by the monitoring system. Table 1 summarizes the characteristics of the most active phylogenetic clusters over a 12 month period. These active clusters tended to be predominantly MSM, with substantial variation among clusters in age distributions and prevalence of transmitted drug resistance.

**Figure 2:**
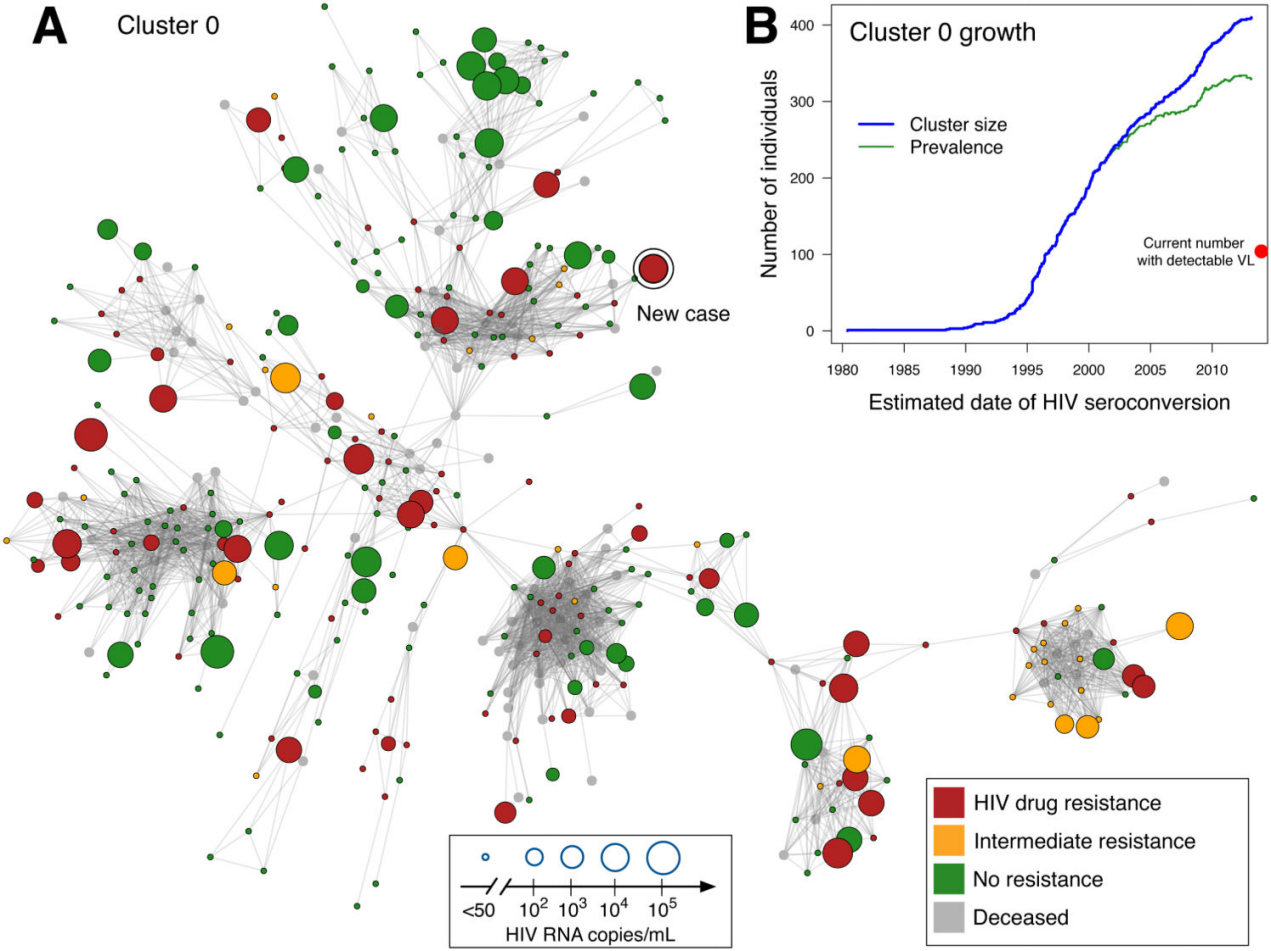
Network diagram and growth trend for cluster index 0 This figure depicts diagrams that are generated for the phylogenetic monitoring reports. The largest phylogenetic cluster from the BC DTP database, cluster 0 is largely composed of people who use injection drugs in the Vancouver ‘Downtown Eastside’. (A) Each circle in the network diagram corresponds to a person living with HIV, sized in proportion to their most recent viral load, and colored to indicate whether any HIV genotype tests for that individual have been classified with high (red) or intermediate (orange) HIV drug resistance, or mortality (grey). This annotation scheme emphasizes parts of the network where transmissions are most likely to occur, notwithstanding individuals who have not yet been sampled. New cases within the reporting period are indicated by a double outline. Lines drawn between circles indicate that the shortest distance separating HIV sequences from the respective individuals in the phylogenies fell within the clustering threshold. (B) The blue line represents the growth of cluster 0 based on the imputed dates of HIV seroconversion. Prevalence (green) was estimated by subtracting deceased cases from the growth trend. A red circle indicates the estimated number of cases for which the most recent viral loads were at detectable levels, based on the available data at the end of the reporting period.

**Figure 3:**
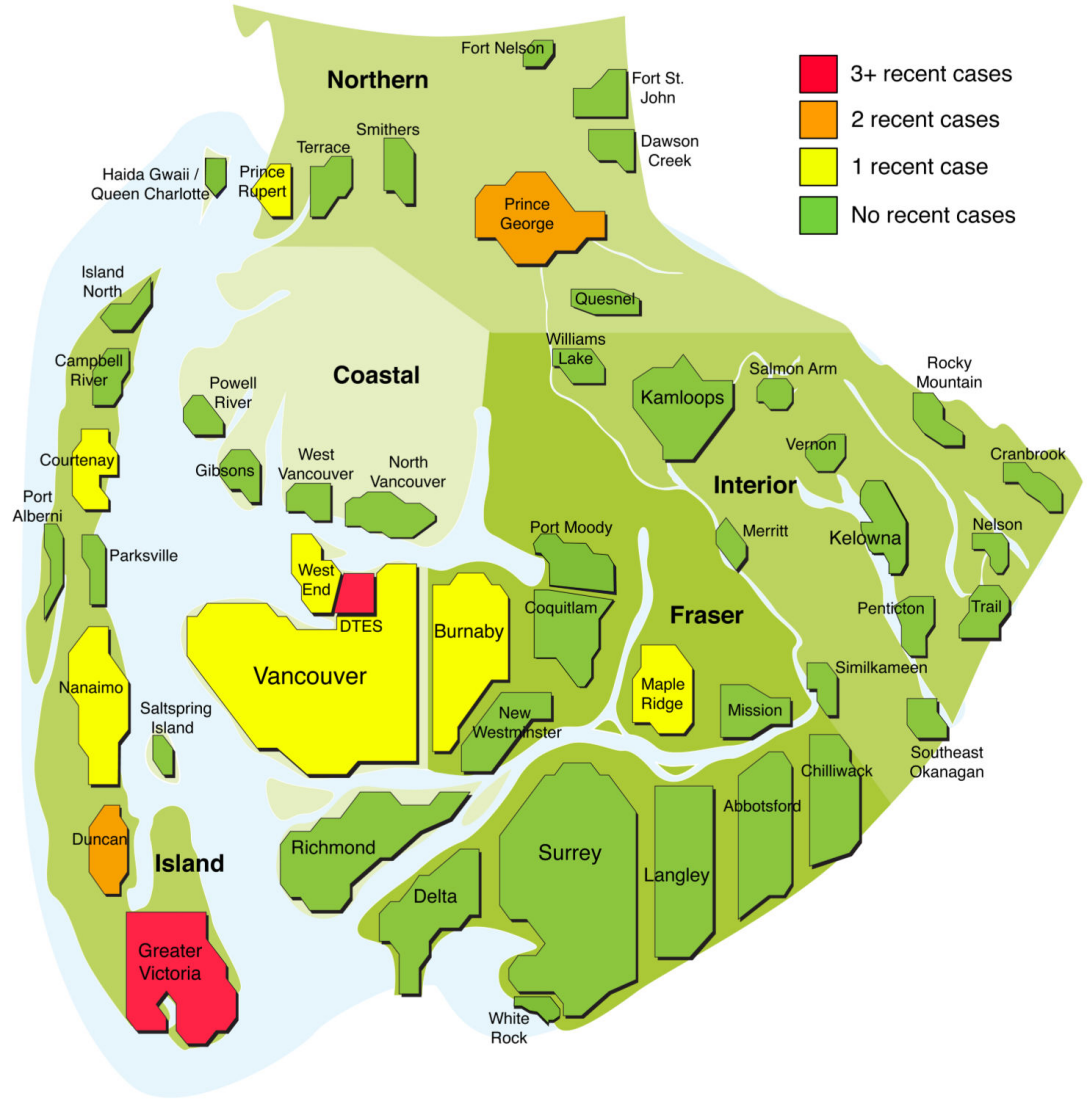
Geographic distribution of cluster 0 This simplified map of the province of BC is automatically generated by the monitoring system for every active cluster within the reporting period. The map, adapted from a cartogram published in The British Columbia Atlas of Child Development^21^, is distorted to emphasize regions with higher population densities. In this example, cases in cluster 0 were defined as recent when their earliest database entry date was in 2014 or later. The distribution of recent cases among regions, based on the forward sortation areas of the physicians' offices where individuals have most recently accessed primary care, is indicated by the coloration of the respective polygons (see figure legend). Bold labels indicate the five regional health authorities of BC.

Figures 2 and 3 provide examples of the diagrams automatically generated by the monitoring system for every active cluster within the reporting period, which are designed to inform the prioritization of clusters for public health actions. For example, nodes in the network diagram are scaled to each individual's most recent plasma viral load. This particular set of diagrams correspond to cluster 0, the largest phylogenetic cluster in BC that largely comprises people who use injection drugs in the Vancouver ‘Downtown Eastside’^23^. Cluster 0 has one of the most widespread distributions of all phylogenetic clusters (Figure 3), due in part to the cluster's size (*n*=414 as of October 8, 2015) and long history of expansion (Figure 2B).

**Figure 4:**
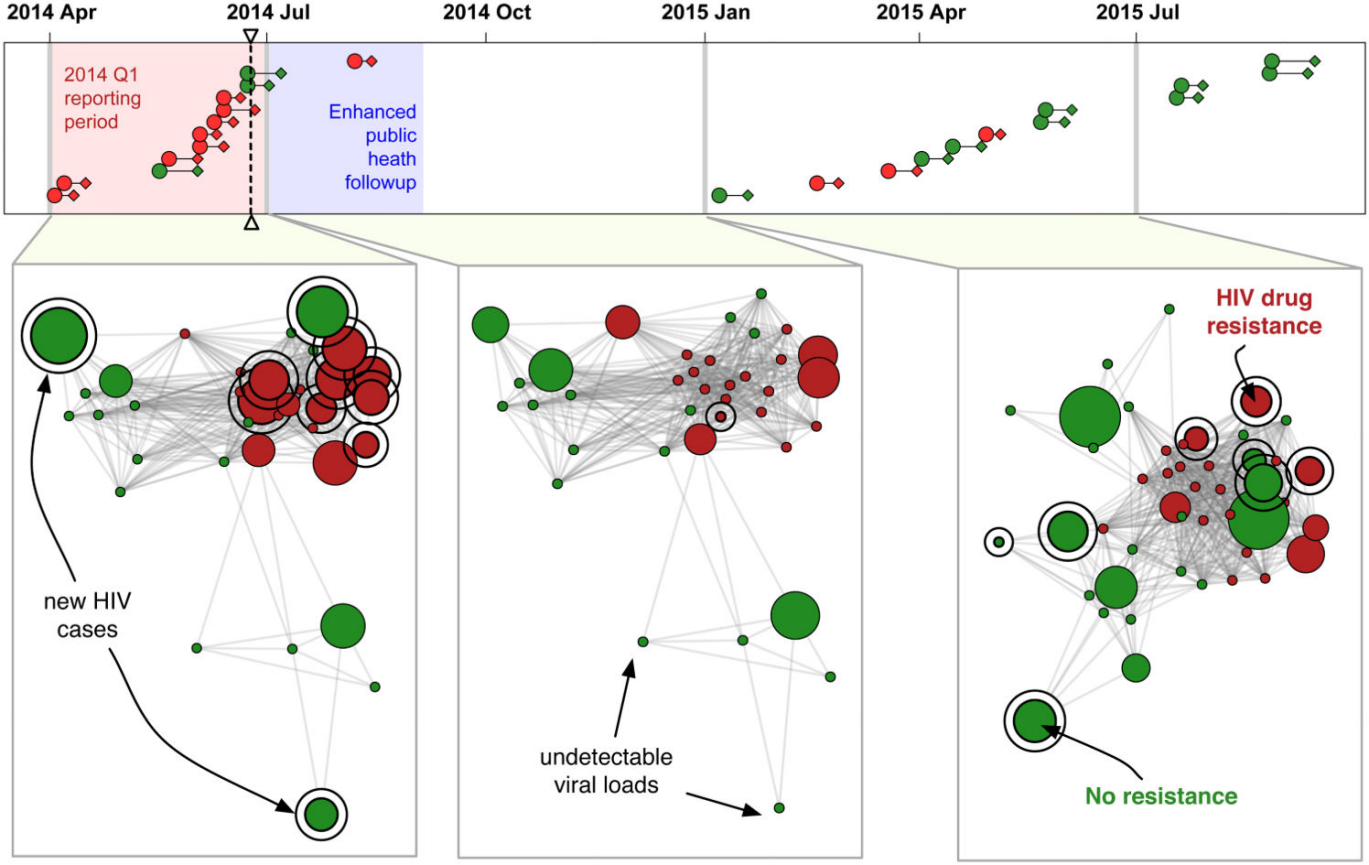
Timeline of cluster 55 outbreak A timeline of the growth of cluster 55 is illustrated by the upper diagram where new cases are mapped to their respective dates of sample collection (circle) and HIV genotype database entry (diamond), and colored with respect to HIV drug resistance. A dashed line indicates the date that a provisional report on an outbreak of 8 new cases in cluster 55 was issued. The network diagrams in each panel below the timeline summarize the phylogenetic relationships among the new cases and their most recent viral loads by the end of the respective reporting periods. Note that the HIV genotype connecting an individual to others in the network and their most recent viral load were not necessarily derived from the same sample.

In June 2014, the monitoring system determined that cluster 55 had grown by 11 new cases over a period of three months relative to the respective sample collection dates (Figure 4, left panel). A total of eight new cases carried the transmitted drug resistance mutation K103N in HIV RT, which confers resistance to first generation non-nucleotide RT inhibitors, and a median viral load of 4.9 log_10_ copies/mL. All but one of the individuals had accessed care within Vancouver Coastal Health (VCH) regional health authority. A provisional report on eight of the 11 new cases in cluster 55 was issued on June 24, 2014 (Figure 4, dashed line), a week in advance of the scheduled quarterly report, to the BCCDC and VCH. The baseline samples from the remaining three new cases had not yet been processed by the date of the provisional report. The rapid spread of transmitted drug resistance identified by this provisional report was deemed a sufficient public health concern that the Provincial Health Officer of British Columbia, under the authority of the Public Health Act, issued a formal request to the Chief Medical Health Officer of VCH to perform a public health investigation of all 36 individuals within cluster 55 who had accessed care within VCH. Because HIV is a reportable disease in British Columbia, the corresponding HIV diagnoses were already known to VCH public health. For the eight new cases that appeared to CFE from April to June 2014, the dates of HIV diagnoses ranged from February to June 2014. Of the 36 individuals, 33 unique individuals were identified (three individuals had laboratory tests requisitioned under two different names), of which 27 were already known to VCH public health, including all new cases with transmitted drug resistance, either in the Sexually Transmitted Infection surveillance database or in the Primary Access Regional Information System. All of the known cases were MSM, with a median age at diagnosis of 27 (range 19 to 56) years, of which 12 (44%) had been diagnosed with acute stage disease, as defined by a laboratory testing algorithm and testing history^24^, substantially greater than the average proportion of acute stage diagnoses in the community since 2012.

During this investigation, an additional new case carrying the transmitted drug resistance mutation had appeared in cluster 55. An enhanced public health follow-up was initiated on July 2, 2014 for the nine new cases in cluster 55 (Figure 4, blue highlight). Two additional cases without HIV drug resistance subsequently appeared in cluster 55 (July 2 and 7) that were not included in this follow-up. The objective of the enhanced follow up was to ensure linkage to care and initiation of ART, and to complete any outstanding partner notification, testing and linkage to care (Appendix, p.4). Prior to July 2, only five of the nine individuals were on treatment, of which only one had attained a suppressed viral load; the median viral load was 38109 copies/mL. By September 5, 2014, eight of the individuals had initiated ART and 6 had attained undetectable viral loads, with a median viral load of <40 copies/mL (below the limit of detection; Figure 4). All nine individuals had received partner counseling and referral services (PCRS) at the time of their HIV diagnosis. During the enhanced follow-up, seven individuals were willing to re-engage with public health for further partner counseling and notification. This resulted in 12 additional contacts (five known, seven anonymous) in addition to the 37 contacts (17 known, 20 anonymous) from the initial PCRS. The number of known contacts who were known to be previously HIV positive increased from two to six individuals. Of these four previously positive contacts, one individual who was named as a contact of an acute case of HIV had been disengaged from care and involved in behaviors with high risk of onward HIV transmission. This individual was re-engaged in care as a result of the enhanced follow-up. Subsequent to the start of the enhanced follow-up, cluster 55 was relatively inactive until the end of 2014 with only one additional case (August 14, 2014; Figure 4, centre panel); however, a total of 12 new cases gradually appeared in the following year, of which three carried drug resistance (Figure 4, right panel).

## Discussion

The enhanced public health follow-up of the cluster 55 outbreak was followed by the suppression of viral loads in most affected individuals and several months without new cases. It is difficult to attribute a causal effect to this intervention since there was no “control” group undergoing a similar outbreak where public health action was withheld. However, a majority (75%) of the 12 new HIV cases that appeared in cluster 55 since January 2015 were non-resistant strains mapping to parts of the HIV phylogeny that were not targeted by the enhanced public health follow-up. This outcome suggests that the follow-up partially fulfilled its primary objective of preventing the onward transmission of HIV drug resistance, though it was not completely successful in preventing further transmissions in the cluster. Although this follow-up concluded in 2014, cluster 55 has remained a focus of public health actions.

The use of routinely collected clinical HIV data to inform public health interventions raises important ethical considerations. We have utilized the ethics framework of public health to determine how this information should be collected and used for HIV prevention measures. The guiding principle of this framework is to use the least intrusive but most effective intervention available. As the use of phylogenetics is an emerging area in public health, there are no best practice guidelines for using this information. HIV is a reportable disease in BC. Under the BC Public Health Act Communicable Disease Regulation, a medical health officer may request personally identifying information from a physician when there is reasonable likelihood of HIV transmission. The primary objective of public health management is to prevent onward transmission of HIV by reaching populations at risk – not to ascribe transmission to specific individuals. Accordingly, we have used the phylogenetic cluster defined at a minimum size of five individuals as an operative unit for public health surveillance. When cluster 55 was deemed a significant public health concern, all individuals in the cluster were identified in accordance with the provisions of the Communicable Disease Regulation. Minimal information for all individuals in the cluster was securely transmitted in an encrypted format to VCH Public Health, which was already aware of all persons diagnosed with HIV in the region. The identification of groups to offer counseling, testing and treatment in rapidly growing phylogenetic clusters is consistent with the aims of HIV prevention, and discourages the attribution of fault with any one individual.

One of the general limitations to monitoring HIV transmission hotspots from routine genotyping is that it is restricted to populations with members who present for HIV testing and have a plasma viral load test. Under current provincial guidelines, a genotype test is automatically performed using the residual blood plasma from the baseline viral load tests for all new HIV diagnoses. We estimate that over half of all residents of BC living with HIV have HIV genotype test records in the DTP database. This sampling density is well within recommended levels for the reproducible identification of clusters^25^. However, individuals who are untested or disengaged from care are likely underrepresented in the database population. A recent meta-analysis suggested that the undiagnosed population may contribute disproportionately to the onward transmission of HIV^26^; hence, the impact of realtime monitoring may be contingent on optimizing all stages in the cascade of HIV care. Furthermore, the time between HIV infection and diagnosis inevitably varies substantially among individuals. Despite these unavoidable limitations, we have demonstrated that phylogenetic monitoring can supplement standard epidemiological methods and inform public health actions on a time scale that is sufficient to potentially alter the course of a localized outbreak.

Another challenge for the implementation of HIV phylogenetic monitoring is finding the balance between protecting an individual's right to privacy and right to refuse medical care, and the public health responsibility to prevent the onward transmission of HIV. This dilemma is exacerbated by the criminalization of HIV exposure^27^ or transmission^28^, since the same phylogenetic methods used for characterizing transmission hotspots have also been misused to prosecute individuals for the transmission of HIV^29^. It is further compounded by the widespread use of the terms “transmission network” or “transmission cluster” to refer to genetically-similar virus populations, which implicitly equates a phylogenetic relationship with a transmission event. On the contrary, a molecular phylogeny cannot establish whether the virus was transmitted directly from one individual to another, due to the extensive diversity of HIV within hosts^6^ and the potential for transmissions through unknown third parties. Furthermore, a phylogeny cannot determine the directionality of HIV transmission without extensive clonal sequencing of the virus populations^30^.

The ubiquity of routine HIV genotyping in developed settings, where the comparably low but highly heterogeneous prevalence of HIV poses significant challenges for the cost-effective deployment of HIV prevention resources, is strong motivation for near real-time monitoring through the secondary analysis of data already being collected as standard of care. We have presented a case where our monitoring system prioritized a specific outbreak of transmitted HIV drug resistance for public health intervention. Such actions will ultimately become important for supporting targeted HIV prevention efforts and preserving treatment options for the population, and may translate to other areas of infectious disease.

## Supplementary Material

1

## Figures and Tables

**Table 1:** Summary statistics of the ten most active clusters from October 2014 to October 2015

Cluster index	New cases	Current size	MSM	IDU	On therapy	TDR	PCS=0	Age ≤ 30y
7	23	99	45 (48) 94%	7 (56) 12%	68 (88) 77%	0 (81)	42 (87) 48%	31 (88) 35%
55	12	49	28 (29) 97%	3 (31) 10%	38 (49) 78%	17 (48) 35%	22 (49) 45%	30 (49) 61%
0	11	414	10 (280) 4%	245 (294) 8%	214 (325) 66%	11 (204) 4%	30 (313) 10%	71 (325) 21%
3	9	86	45 (51) 88%	9 (57) 16%	65 (79) 82%	1 (76) 1%	25 (78) 32%	12 (84) 14%
1	7	84	48 (52) 92%	10 (59) 17%	64 (78) 82%	1 (78) 1%	22 (78) 28%	9 (79) 11%
203	7	9	9 (9) 100%	0 (9)	8 (9) 89%	8 (8) 100%	3 (8) 38%	6 (9) 67%
49	5	26	11 (12) 92%	3 (12) 25%	21 (24) 88%	0 (23)	15 (24) 62%	11 (24) 46%
217	5	8	4 (5) 80%	1 (7) 14%	8 (8) 100%	0 (8)	0 (8)	4 (8) 50%
17	4	48	24 (30) 80%	6 (34) 18%	31 (46) 67%	0 (43)	7 (44) 16%	5 (45) 11%
200	4	18	8 (10) 80%	1 (13) 8%	10 (15) 67%	0 (13)	1 (14) 7%	0 (15)

We report the numerator, denominator (in parentheses) and percentage for the following attributes: MSM = individuals who self-reported as men who have sex with men; IDU = individuals who self-reported as having ever used injection drugs; TDR = individuals carrying one or more transmitted HIV drug resistance mutations prior to the start of ART; “PCS=0” = individuals with a programmatic compliance score of 0, indicating complete adherence to the International Antiviral Society-USA treatment guidelines^17^; “Age≤30y” = individuals whose age at first sample collection date was 30 years or less.

## Panel: Research in context

### Evidence before this study

We queried the Google Scholar database for similar studies using the search terms “HIV”, “genotype”, “clustering” and “monitoring” with no date limits. Phylogenetic trees reconstructed from HIV sequence data have been shown to bear similar shapes to recent transmission histories of the virus owing to its rapid evolution, even in the relatively conserved regions of its genome that encode the primary targets of antiretroviral treatment. Furthermore, a number of groups have demonstrated the utility of clustering methods applied to HIV sequence data to retrospectively characterize the variation in rates of HIV transmission among subpopulations in association with risk factors. This prior evidence implies that routinely-collected HIV genotype data can be used to track the progress of an epidemic in near real time.

### Added value of this study

We have implemented an automated HIV monitoring system in British Columbia, Canada, that utilizes the provincial HIV drug treatment population database to track the growth of phylogenetic clusters of closely-related HIV infections in near real time. Here, we report a case study of the utilization of reports from this monitoring system in direct support of an HIV outbreak investigation and enhanced public health follow-up, which was associated with a marked reduction in transmitted HIV drug resistance in the affected subpopulation.

### Implications of all the available evidence

The evidence provided by this study and previous research imply that near real time monitoring based on the clustering analysis of routinely-collected HIV genotypes can become an effective and cost-effective resource for public health intervention on localized outbreaks of HIV transmission.
