# Supplementary material for: Near real-time monitoring of HIV transmission hotspots from routine HIV genotyping: an implementation case study
Source: Lancet HIV. Author manuscript; Available in PMC 2017 May 1. (PMC4853759; doi:10.1016/S2352-3018(16)00046-1)

Near real-time monitoring of HIV transmission hotspots from routine HIV genotyping: an implementation case study

Supplementary Appendix

Supplementary Text S1. Imputation of HIV seroconversion dates

Physician reports supply estimated dates of HIV seroconversion for roughly 50% of all individuals enrolled in the British Columbia Drug Treatment Program (DTP). We used a hot-deck multiple imputation method to impute the estimated seroconversion date (t_S) for all individuals missing this variable. For each case X in which t_S was missing, we used the following procedure:

1. A "complete" case Y with known t_S and the same risk factor profile as X was selected at random from the data set.
2. The difference Δt between the baseline sample collection dates for X and Y was calculated in years.
3. A random number U was drawn from a uniform distribution between 0 and 1.
4. If U was less than the probability density at Δt for a normal distribution with mean 0 and standard deviation of 0.5 years, then we proceeded to step 6.
5. Otherwise, we returned to step 1.
6. Given Y, we calculated the difference ΔT between its baseline sample collection date and t_S.
7. The estimated seroconversion date for X was set to $t - \Delta T$, where t was the baseline sample collection date for X.

This procedure was repeated ten times and the imputed dates were averaged to obtain the final estimate for case X.

Supplementary Text S2. HIV genotyping

The BC Centre for Excellence in HIV/AIDS (CFE) Molecular Laboratory program follows a set of internally developed and validated standard operating procedures for performing HIV genotype tests for the BC Drug Treatment Program. Quality control of genotyping is largely implemented by a software program (RECall[1]) that was developed in-house to automate the base-calling and assembly procedure in the conversion of chromatograms into sequence. RECall aligns chromatograms against a defined reference sequence and identifies ambiguous bases (mixtures) based on the quality and area under the curve of the called and uncalled bases. It also marks potentially problematic sequences according to a set of internal quality metrics that have been calibrated from expert operators; the respective positions are flagged to be manually reviewed by the user. By default, RECall requires a minimum of two-fold coverage at all positions of the sequence. RECall also automatically screens sequences for evidence of hypermutation.

All sequences from a given run on the ABI 3730xl DNA Analyzer (Applied Biosystems, Foster City, California, USA) are compared to lab reference strains (such as HXB2 and NL4-3) to screen for contaminants. Every run includes at least one internal control sample that consists of either a clonal population of NL4-3 or a known patient sample. The sequence from the control sample must match every other sequence that has ever been generated from that control. A phylogenetic tree is generated using a distance-based method for all samples in a run, along with sequences from all previous samples from the respective patients in the run, to ensure that sequences from the same patient are clustered in the tree. In addition, Hamming distances are calculated for all samples within the run, along with all sequences generated in the past 30 days, to ensure that each sample is genetically distinct. All sequences are screened for premature stop codons and subtyped by calculating genetic distances to predefined subtype reference sequences.

The majority of HIV genotypes (n = 27850, 86%) covered 1497 bases of HIV *pol* encoding protease and the first 400 codons of reverse transcriptase (RT). An additional n = 3946 genotypes were derived from a shorter amplification product covering 1017 bp of *pol* encoding protease and the first 240 codons of RT. Finally, n = 709 genotypes were derived from three partial *pol* sequences of lengths 276, 297, and 363 bases respectively, to cover protease and RT codons 24 to 236 inclusive. Forty-one sequences that contained ambiguous base calls at more than 5% of nucleotide positions were excluded from further analysis, because these sequence ambiguities can induce spurious clustering[2].

Supplementary Text S3. Bootstrapping

Bootstrap resampling is a non-parametric method from computational statistics for quantifying the uncertainty in parameter estimation when replication is not feasible. Instead of performing additional experiments in the real world, we take the existing data set and sample observations at random with replacement to generate artificial data sets with the same number of observations. In phylogenetics, bootstraps are generated by sampling columns of the actual multiple sequence alignment to yield new alignments with the same dimensions (number of observations and positions)[3]. A phylogeny is reconstructed from each bootstrap alignment. The proportion of bootstrap phylogenies in which a specific monophyletic clade appears (a specific set of taxa related by a common ancestor to the exclusion of all other tips) is used to quantify our level of uncertainty in that clade, and is known as the bootstrap support value.

Our application of bootstrapping for defining phylogenetic clusters is significantly different from the estimation of bootstrap support values because our objective is to estimate the mean patristic distance between tips. Precision in estimation of this mean as quantified by the standard error has diminishing returns with increasing sample size. Put another way, expanding the number of bootstrap replicates from 100 to 1000 is expected to increase our precision by the same factor as increasing from 10 to 100. This is not an adequate incentive for increasing the computing time of this processing stage from two to 20 hours. To illustrate, we generated plots of the mean patristic distance with increasing sample size for an arbitrary selection of pairs of tips in bootstrap replicate trees:

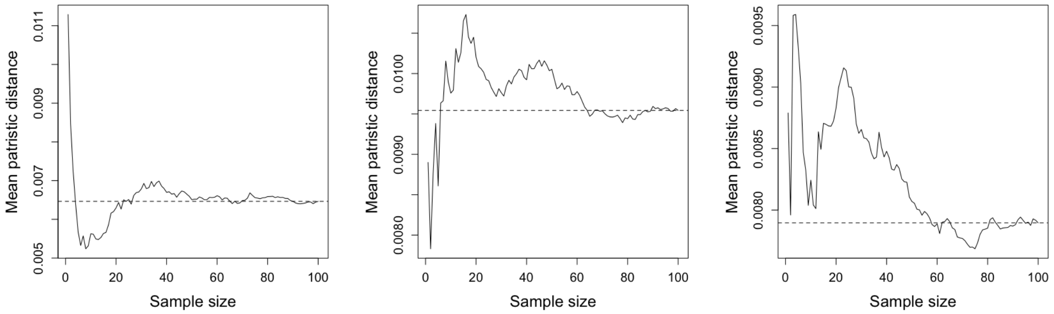

The dashed lines indicate the empirical mean for all 100 bootstrap replicates. Note that the vertical scale varies among plots to accommodate the respective ranges in the estimated means. Furthermore, the key parameters to estimate are the cluster memberships of individuals in the population, which rarely depends on a single patristic distance measure. In other words, individuals are usually connected to their peers in a cluster by multiple edges that represent short patristic distances. Thus, the assignment of individuals to clusters should be more robust to variation in the reconstruction of path lengths among bootstrap phylogenies.

Supplementary Text S4. Phylogenetic reconstruction

A phylogeny is a tree-based model of the ancestral relationships among populations. In this context, the tree represents how the virus populations in different individuals are related through common ancestors. Phylogenetic trees were generated from bootstrap sample alignments using the software program FastTree2 [4]. FastTree2 implements an approximate maximum likelihood heuristic algorithm to reconstruct a tree from the multiple sequence alignment. The overall objective of phylogenetic reconstruction by maximum likelihood is to search the space of all possible trees to find the tree that does the best job explaining the observed variation among sequences. First, it generates an initial tree topology using a fast neighbor-joining method that is based on a custom memory-efficient distance metric that does not adjust for multiple hits. Next, FastTree2 attempts to reduce the total length of the tree (equivalent to a minimum evolution criterion) by rearranging its topology using a limited number of nearest-neighbour interchange and subtree-prune-regraft operations. During this phase, branch lengths are derived from the Jukes-Cantor genetic distance that assumes equal nucleotide frequencies and substitution rates. Finally, it performs a maximum likelihood heuristic search using only nearest-neighbour interchange operations; branch lengths were re-estimated by maximum likelihood under the default generalized time-reversible model of nucleotide substitution[5]. We provide a visualization of one of the resulting phylogenetic trees as Supplementary Figure S1, below.

Supplementary Text S5. Patristic distances

We used a custom tree-traversal algorithm to efficiently extract patristic distances from a phylogeny. This algorithm was implemented in Python using the BioPython Phylo module to parse Newick tree strings. Simply put, we start from a tip of the tree, and then step down to its ancestor and back up to the next descendant. If that descendant is the ancestor of other nodes, then we move up each branch in turn. On the other hand, if the descendant is also a tip, then we move back down two nodes to the next ancestor and repeat the process. Throughout this traversal of the tree, we track the total path length to the original tip. If the path length ever exceeds our cutoff, then we stop traversing the tree along that path.

For each individual, we located the tip in the phylogeny corresponding to the earliest available sequence. Starting with this tip (terminal node) as X, we used a recursive procedure to find all tips within a maximum patristic distance in the tree, as described by the following pseudocode:

```
Define X to be the current node.
Define L to be the total path length.
Define L* to be the maximum path length.
Define {tips} as a list of all tips within a path length L* of the starting node, and their
respective path lengths.

Function stepup ({tips}, X, L):
        Add the branch length of X to L.
        If L is greater than L*, return.
        If X is a tip, then add (X, L) to {tips} and return.
        Otherwise, call stepup for every descendant of X and then return.

For each individual:
        Set L to 0.
        Let X be the tip corresponding to that individual's earliest sequence.
        Add the branch length of X to L.
        Let par(X) be the "parent" node immediately ancestral to X.
        For every descendant Y of par(X) other than X:
                Let L_Y be the branch length of Y.
                If Y is a tip and L + L_Y is less than L*, add (Y, L + L_Y) to {tips}.
                Otherwise, call function stepup({tips}, Y, L).
        While par(X) is not the root:
                Move down the tree by setting X to par(X).
                Add the branch length of X to L.
                If L is greater than L*, then stop.
                For every descendant Y of par(X) other than X:
                        Let L_Y be the branch length of Y.
                        If Y is a tip and L + L_Y is less than L*, add (Y, L + L_Y) to {tips}.
                        Otherwise, call function stepup({tips}, Y, L).
```

Supplementary Text S6. Cluster indexing

As one of several measures to maintain data privacy, the anonymized patient labels in the Drug Treatment Database were automatically replaced with a new set of randomized labels by the monitoring system with every database transaction. Furthermore, all sequences and phylogenetic trees were automatically securely erased from the filesystem immediately following the generation of a report. One of the major challenges that resulted from these security measures was to enable the system to recognize a phylogenetic cluster as the same cluster that appeared in a previous analysis. We used the sequence of the five earliest baseline sample collection dates (in ascending order) of individuals in a cluster as a persistent identifier of that cluster that was recorded in a file. The register of clusters was initiated on July 31, 2013, at which time a total of 155 clusters were assigned integer indices in descending order with respect to their size (number of individuals).

Whenever the system extracts clusters from bootstrap phylogenies, it generates the collection date sequence for each cluster and compares this sequence to the sequences in the persistent register. If there is no exact match in the register, then the system searches for the closest match within a tolerance of two mismatched dates. If no inexact match is found, then the cluster is recorded into the register as a new cluster and assigned an index by incrementing the largest existing index by one.

Supplementary Text S7. Partner notification and counseling

The best practice standards for partner notification in the context of HIV counseling and follow-up in British Columbia[6] include the following: (1) Partner notification services must be available for all newly infected persons; (2) all information disclosed is strictly confidential and participation is always voluntary; (3) the public health nurse (PHN) must explain to the client the options for informing partners and assist the client in deciding the best plan for reaching each partner confidentially and referring them to counselling, testing, and other support services; (4) the PHN and client must prioritize reaching partners based on who is most likely to transmit infection to others and who is most likely to have become infected; (5) locating and notifying activities must begin promptly once the plan for reaching partners has been formulated; (6) the PHN must use standardized data collection tools throughout to maintain the privacy and confidentiality of the client and their partners.

The enhanced public health follow-up on cluster 55 adhered to these same best practices for partner referral and counseling, which are offered for every newly diagnosed individual in BC including those in cluster 55. The enhanced follow-up was initiated on July 2, 2014, for nine cases that recently appeared in the BC Drug Treatment Database in association with cluster 55. The purpose of the follow-up was to ensure linkage to care and treatment initiation, and to complete any outstanding partner notification, testing, and linkage to care. As of September 5, 2014, eight of the clients had initiated antiretroviral treatment with six achieving viral suppression. All nine clients had already received partner counseling and referral services at the time of their HIV diagnosis. After July 2, 2014, seven clients were willing to re-engage with public health for further partner counseling and notification. This resulted in the identification of 12 additional contacts (five known and seven anonymous contacts) for a total of 49 contacts (22 known, 27 anonymous). Among the 22 known contacts, six were determined to be previously HIV positive, and seven who were not previously positive were tested for HIV (Table S1).

Supplementary Table S1 – Outcomes of additional partner notification and counseling.

Partner counseling and referral services (PCRS) were provided to individuals in cluster 55 at HIV diagnosis prior to the initiation of an enhanced public health follow-up (July 2, 2014), and again in the subsequent period as a direct result of the enhanced follow-up.

**PCRS Indicator**	**Before July 2, 2014**		**After July 2, 2014**	
	N	%	*N*	%
Total number of contacts elicited	37		49	
Anonymous contacts	20	54	27	55
Known contacts	17	46	22	45
Known contacts notified	14	82	20	91
Previously known to be HIV+	2	14	6	30
Previously HIV+ reengaged in care	0	0	1	2
Tested for HIV	5	42	7	50

Supplementary Figure S1. Approximate maximum likelihood phylogeny of HIV genotypes. This phylogenetic tree was generated using the baseline HIV genotype records of all individuals in the BC Drug Treatment Database on October 8, 2015. None of the tips are labeled to maintain data privacy. The tree was generated from the aligned HIV-1 *pol* sequences using the approximate maximum likelihood heuristic implemented in the program FastTree2 (version 2.1.7) under the default generalized time reversible model of nucleotide substitution. Branch lengths in this tree are scaled in units of expected numbers of nucleotide substitutions (see scale bar). Subsequently, the tree was rooted on the longest branch separating subtype B and non-B subtype sequences. Branches corresponding to sequences that were placed in phylogenetic clusters by our patristic distance-based algorithm (see Supplementary Text S5) are coloured with respect to the five most active clusters in the period spanning October 2014 to October 2015.

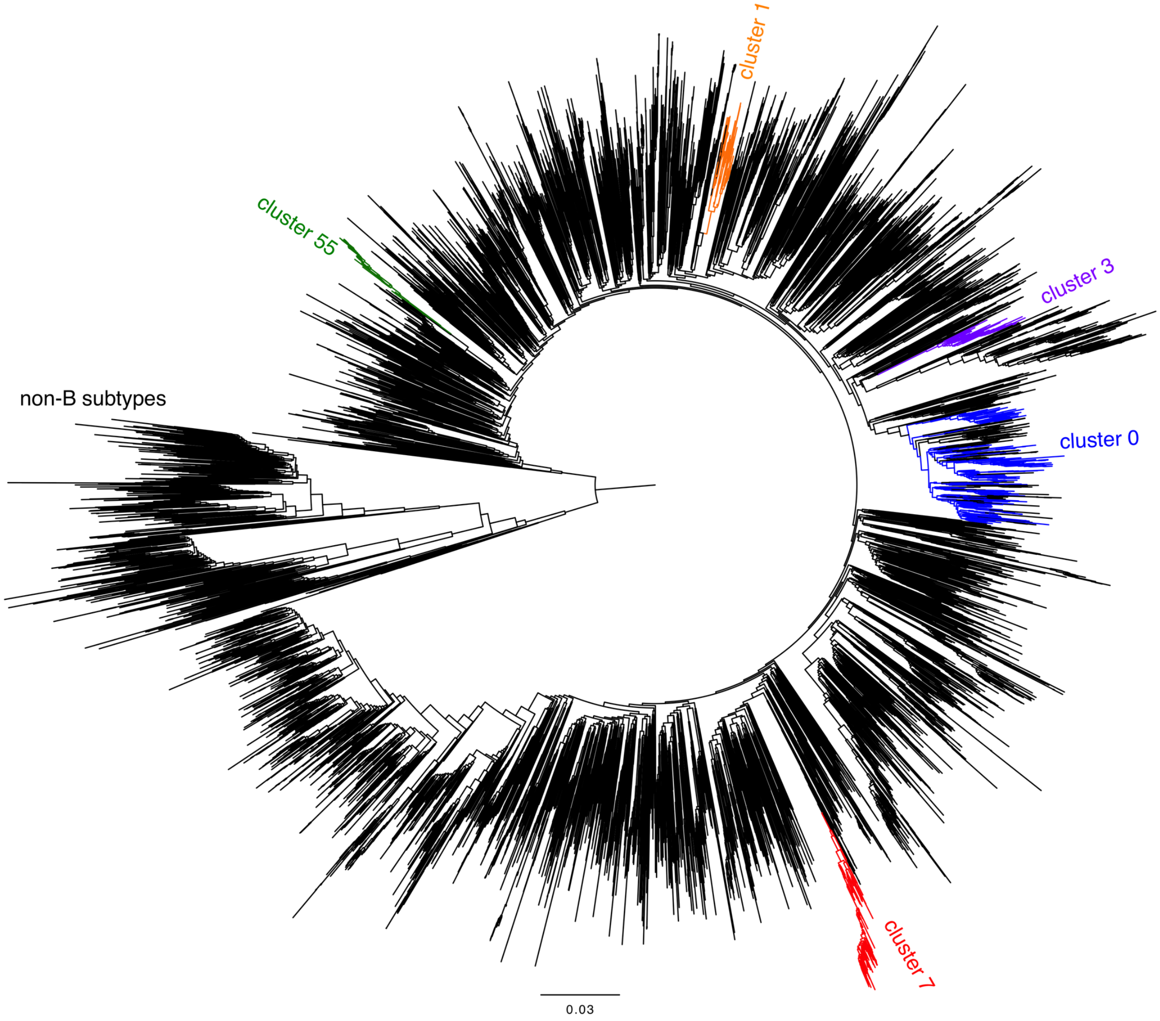

References

1. Woods CK, Brumme CJ, Liu TF, et al. Automating HIV drug resistance genotyping with RECall, a freely accessible sequence analysis tool. *J Clin Microbiol* 2012; **50**(6): 1936-42.
2. Wertheim JO, Leigh Brown AJ, Hepler NL, et al. The global transmission network of HIV-1. *The Journal of infectious diseases* 2014; **209**(2): 304-13.
3. Felsenstein J. Confidence-Limits on Phylogenies - an Approach Using the Bootstrap. *Evolution* 1985; **39**(4): 783-91.
4. Price MN, Dehal PS, Arkin AP. FastTree 2--approximately maximum-likelihood trees for large alignments. *PloS one* 2010; **5**(3): e9490.

5. Tavaré S. Some probabilistic and statistical problems in the analysis of DNA sequences. *Lectures on Mathematics in the Life Sciences* 1986; **17**: 57-86.
6. BC Centre for Disease Control. Communicable Disease Control: HIV-1, HIV-2 Counselling and Follow Up Policy. 2007. http://www.bccdc.ca/resource-gallery/Documents/Communicable-Disease-Manual/Chapter 5 - STI/STI_Policy_HIV1_2_20071001.pdf.

HIV Phylogenetic Monitoring Report

January 20XX -- MOCKUP

BRITISH COLUMBIA
CENTRE *for* EXCELLENCE
in HIV/AIDS

Contents

Pretext 2
Background 2
Phylogenetics 2
Application to HIV epidemiology 2
Abbreviations 2
Database population 3
Summary statistics 3
Breakdown by health authority 3
Cluster analysis 4
Composition by clinical and risk factors 4
Composition by BC Health Authority 5
Composition by programmatic compliance 6
Detailed Cluster Reports 7
Network diagrams 7
Growth trends 7
Age distributions 7
Distribution by health authority 7
Geographic distribution 7

Pretext

This document was prepared by the British Columbia (BC) Centre for Excellence in HIV/AIDS Laboratory Program for limited distribution to members and affiliates of the BC Centre for Disease Control and the Ministry of Health. ~~**Public release or redistribution of this document or any of its contents is prohibited.**~~

This is a mockup version of the monthly report. All variables have been randomized.

Background

Phylogenetics

A **phylogeny** is a tree-based model of how populations are related by common ancestors. A molecular phylogeny is reconstructed from genetic sequence data. In the context of virus populations, each genetic sequence represents the consensus sequence of the entire virus population within a given patient. Each `tip' of a phylogeny corresponds to a genetic sequence. The tips are joined by branches that represent lineages of descent from common ancestors.

Application to HIV epidemiology

The evolution and transmission of HIV unfold on similar time scales. A virus population can accumulate new mutations within months of infection, making it genetically unique to that patient. Encountering infections that remain genetically similar implies that those virus populations are related by one or more recent transmission events. While the possible interpretations include the transmission of the virus from one individual to another, it is impossible to determine which direction the transmission occurred between a pair of individuals. It is also impossible to rule out the possibility that both individuals were infected by a third individual. Nevertheless, we can use this similarity to identify subgroups of the population experiencing higher rates of HIV transmission. By reconstructing phylogenies from HIV sequence data collected for resistance testing, we can locate these `clusters' of elevated transmission rates and characterize the corresponding subgroups.

Abbreviations

FSA = forward sortation area
IDU = injection drug user
MSM = men who have sex with men

Database population

Summary statistics

Index	Description	Records	Individuals (deceased)
A.	Number in database	52000	9888 (742)
B.	Number of *A* new this month	155	182
C.	Number of *B* that appear in clusters	74	20
D.	Number of *C* that represent new cases	12	8

Breakdown by health authority

This table reports the three forward sortation areas (FSAs) with the largest number of cases within each regional health authority. The following proportions are reported for these FSAs: %Unsep = Unsuppressed. %NESup = Proportion not ever suppressed. %NERx = Proportion not ever on therapy. %>6moRx = Proportion with over 6 months on therapy.

Region	#Cases	%Unsup	% NESup	% NERx	%>6moRx
**Coastal**					
Downtown Vancouver, SW Downtown	2232	15	11	12	66
Downtown Eastside, Strathcona	538	27	10	19	85
Vancouver, West Fairview	524	12	9	6	172
Richmond, North	561	13	5	7	98
Downtown Vancouver, SE West End	245	14	14	7	108
**Fraser**					
Surrey, North	78	44	4	3	205
Surrey, Outer Northwest	113	17	7	10	93
New Westminster, Northeast	66	19	10	4	33
Surrey, Inner Northwest	73	19	4	7	38
Maple Ridge, West	79	18	15	7	96
**Interior**					
Kelowna, Central	248	22	13	36	80
Trail,	116	17	4	7	77
Kamloops, Central and Southeast	53	34	16	6	88
Vernon, Central	35	63	10	6	65
Nelson,	13	32	13	9	138
**Island**					
Nanaimo, South	212	19	11	5	115
Greater Victoria, North Oak Bay	254	32	4	2	79
Greater Victoria, Downtown	142	37	13	11	32
Greater Victoria, Rock Bay	55	19	7	16	62
North Vancouver Islan...,	21	38	15	7	76
**Northern**					
Prince George, East Central	81	21	25	25	84
Omineca and Yellowhead,	75	88	24	7	61
Prince George, West Central	44	18	16	7	240
Prince Rupert,	41	38	22	1	135
Terrace,	5	29	5	10	30

Cluster analysis

Composition by clinical and risk factors

Composition of all phylogenetic clusters containing 5 or more individuals. Numbers in parentheses indicate the prevalence (%) of the corresponding factor in the cluster after adjusting for missing observations. **TDR** = transmitted drug resistance, defined as the presence of HIV mutations conferring resistance to one or more drug classes (as determined by the vircoTYPE algorithm). **MSM** = men who have sex with men. **IDU** = injection drug user. **HCV** = co-infection with hepatitis C virus.

On therapy indicates that the patient has picked up their antiretroviral prescription within the current month. **Undetectable** = most recent viral load below limit of detection for the respective assay. The percentages reported for these two quantities are calculated relative to the number of individuals in each cluster who are currently still alive (**Alive**).

Index	Total	Alive	TDR	MSM	IDU	HCV	On therapy	Undetectable
14	124	42		14 (62%)	6 (15%)	3 (18%)	43 (120%)	56 (77%)
62	76	38	22 (58%)	21 (105%)	4 (2%)	4 (14%)	43 (97%)	46 (50%)
105	11	6	2 (18%)	2 (24%)	4 (42%)	3 (115%)	3 (106%)	2 (51%)
71	13	6		5 (100%)	2 (24%)	1 (13%)	10 (98%)	4 (57%)
212	14	4		3 (99%)			8 (64%)	11 (39%)
144	17	24	9 (75%)	7 (110%)			12 (219%)	5 (68%)

Composition by BC Health Authority

For all phylogenetic clusters of size greater than 5, we tabulated all clusters in which new cases emerged this current month with respect to health authority according to the FSA of the physician office requesting the HIV resistance genotype test. If this FSA was not available in the database then the corresponding member of a cluster was categorized as `unknown'. *Coastal* = Vancouver Coastal; *Island* = Vancouver Island.

Index	Total	New	Coastal				Island				Fraser			
			total	6mo	3mo	new	total	6mo	3mo	new	total	6mo	3mo	new
65	66	2	55	**7**	**3**	**1**	1	**1**	**1**	**1**	3	**1**		
32	29	1	25	**1**	**2**		12	**1**	**1**	**1**	1			
54	16	1	9				1				1	**1**	**1**	**2**
188	17	1	3				9	**1**	**1**	**2**	2			
122	11	1	4	**1**	**1**	**1**	1				2			
180	8	1	1				1				1			

Index	Total	New	Interior				Northern				*Unknown*			
			total	6mo	3mo	new	total	6mo	3mo	new	total	6mo	3mo	new
28	87	2	1				0				2			
29	52	1	3				1				3			
30	8	1	0				1				1			
123	14	1	1				1				1			
79	13	1	1				2				1			
236	24	1	10	**14**	**5**	**2**	1				1			

Composition by programmatic compliance

The programmatic compliance score (PCS) is an integer value from 0 to 6 that summarizes compliance to the HIV treatment guidelines of the International AIDS Society (IAS), such as initiating antiretroviral therapy with baseline CD4 cell count below 200 cells/mL. A lower PCS value indicates greater compliance to guidelines.

Index	0 (high)	1	2	3	4	5	6
All	1096 (14%)	688 (22%)	3508 (16%)	1336 (26%)	1460 (9%)	613 (27%)	453 (20%)
22	6 (16%)	21 (26%)	14 (43%)	18 (28%)		3 (12%)	
64	23 (42%)	22 (14%)	9 (17%)	2 (3%)		7 (11%)	
58	4 (18%)	1 (12%)	2 (30%)			1 (4%)	
365	1 (21%)	2 (19%)	3 (49%)		1 (14%)		
132	2 (84%)		3 (54%)				
177	4 (25%)	2 (67%)	1 (6%)	6 (18%)	1 (6%)		

Detailed Cluster Reports

In the following pages, network diagrams, growth trends, distributions of age at baseline, and geographic location (as forward sorting area of physician office) are provided for each cluster in which one or more new cases have occurred in this month.

Network diagrams

Each network diagram in this section depicts a phylogenetic cluster that has grown by one or more new HIV cases in January 20XX. Each `node' in the network represents an HIV-infected individual in BC. Nodes are coloured according to the presence of HIV mutations conferring resistance (red) or intermediate resistance (yellow) to one or more drug classes in ANY sample EVER collected from the respective individual. Otherwise, the node is coloured green to indicate that the infection is susceptible to all three drug classes (protease inhibitors and non-/nucleoside reverse transcriptase inhibitors). Nodes representing new cases are indicated by a double-outline. The size of the node is scaled to the most current plasma viral load measurement, such that larger nodes represent infections with higher viral loads. Nodes corresponding to samples with undetectable viral loads ($<$ 50 copies/mL) are rendered at an even smaller size.

Growth trends

Each `growth trend' figure illustrates the accumulation of individuals over time (based on estimated or imputed dates of HIV seroconversion), for each phylogenetic cluster (blue line). A second trend approximates the number of surviving individuals in each cluster over time, based on mortality data.

Age distributions

For each cluster, the age of each individual at baseline was estimated by the difference between their year of birth (if available) and the collection date of their earliest sample. The age distribution of a cluster is reported by grouping individuals in the cluster into five age classes that would contain roughly equal numbers from the entire population.

Distribution by health authority

The distribution of individuals in each cluster with respect to provincial health authority was determined on the basis of the forward sortation area (FSA, first three digits of the postal code) of the physician offices that had requested the respective HIV resistance genotype tests. Each FSA was mapped to provincial health authorities on the basis of the current list of urban and rural delivery areas published by Canada Post (https://www.canadapost.ca/cpc2/addrm/hh/current/indexc/tcBCu-e.asp).

Geographic distribution

Each cluster was mapped to the FSAs, shown respectively for the Greater Vancouver area and British Columbia (BC), corresponding to the physician offices that had requested the respective genotype tests. Each FSA was coloured with respect to the number of new cases in the past 6 months (increasing intensities of red) or the number of past cases in the cluster (increasing intensities of blue). A colour legend is provided in each BC plot.

Cluster 0

Network diagram of cluster 0

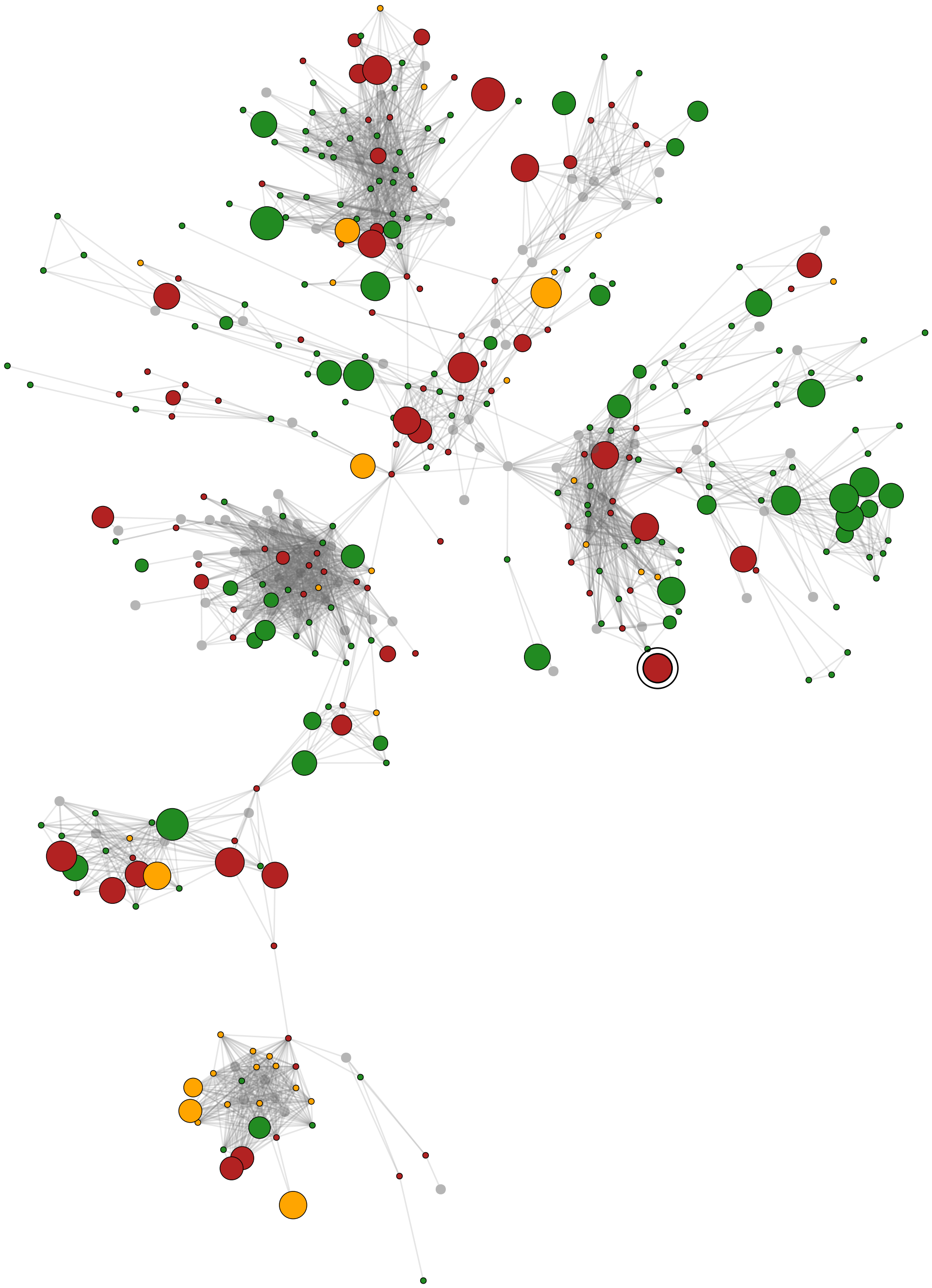

	Resistance to ≥1 drug classes	Total size of cluster	51
	Intermediate resistance to ≥1 drug classes	Number of surviving individuals	32
	Susceptible to all drug classes	Number surviving with current detectable VL	15
	Deceased		
◎	New cases indicated by double circle		

Growth trends of cluster 0

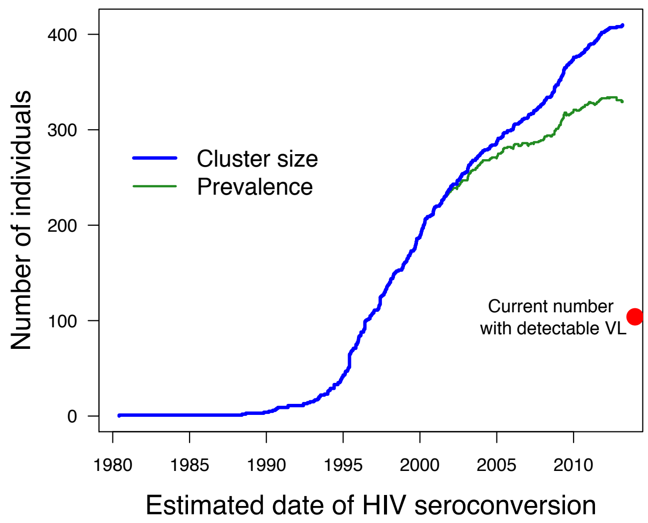

Blue line indicates cumulative number of cases in the cluster.
Green line indicates the number of surviving cases in the cluster (adjusted for mortality, *e.g.*, prevalence). The current number of surviving individuals with detectable viral loads is indicated by a large red circle on the right margin of the plot.

Age distributions of cluster 0

Age group	Total in cluster	Unsuppressed
1 - 37	3	1
28 - 28	6	3
57 - 25	18	1
74 - 74	6	2
26 - 120	21	1

Distribution by health authority of cluster 0

Health authority	Number of individuals	Unsuppressed
Northern	1	2
Coastal	12	6
unknown	1	1
Interior	2	1
Island	21	3
Fraser	1	3

Geographic distribution of cluster 0

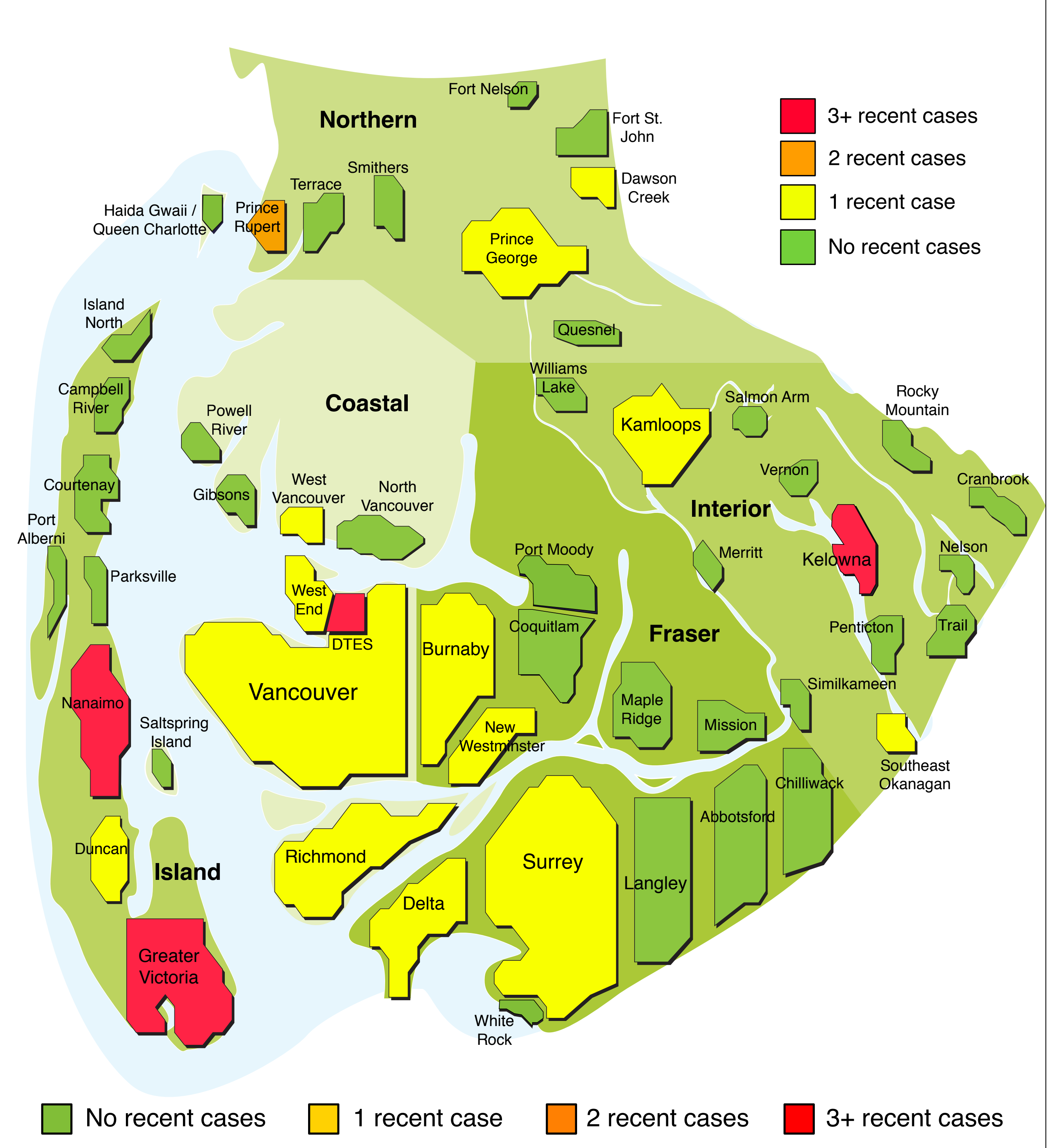

Supplement: 1 [file NIHMS778624-supplement-1.pdf]
